# 4-Bromo-2-({4-[(hy­droxy­imino)­meth­yl]phen­yl}imino­meth­yl)phenol

**DOI:** 10.1107/S160053681002698X

**Published:** 2010-07-14

**Authors:** Yu-Hua Yang, Jian-Chao Wu, Shang-Sheng Gong, Jiu-Si Wang

**Affiliations:** a School of Chemical and Biological Engineering, Lanzhou Jiaotong University, Lanzhou 730070, People’s Republic of China

## Abstract

In the title compound, C_14_H_11_BrN_2_O_2_, the mean planes of the two benzene rings are almost parallel to each other, making a dihedral angle of 4.09 (1)°. An intra­molecular O—H⋯N hydrogen bond occurs. In the crystal, inter­molecular O—H⋯N and C—H⋯O hydrogen bonds link the mol­ecules into a chain-like supra­molecular structure.

## Related literature

For background to the use of Schiff bases as ligands in coord­ination chemistry, see: Biswas *et al.* (2008 ▶); Dong *et al.* (2010 ▶). For the synthesis of the title compound and related structures, see: Dong *et al.* (2007 ▶, 2009 ▶); Zhao *et al.* (2009 ▶).
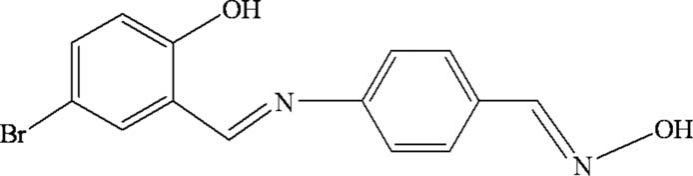


## Experimental

### 

#### Crystal data

C_14_H_11_BrN_2_O_2_
*M* *_r_* = 319.16
Orthorhombic, 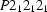

*a* = 4.4279 (5) Å
*b* = 12.1790 (16) Å
*c* = 23.196 (2) Å
*V* = 1250.9 (3) Å^3^
*Z* = 4
Mo *K*α radiation
μ = 3.29 mm^−1^
*T* = 113 K
0.26 × 0.24 × 0.22 mm

#### Data collection

Rigaku Saturn724 CCD diffractometer
Absorption correction: multi-scan (*CrystalClear*; Rigaku/MSC, 2009 ▶) *T*_min_ = 0.482, *T*_max_ = 0.532
15336 measured reflections
4346 independent reflections
2818 reflections with *I* > 2σ(*I*)
*R*_int_ = 0.048

#### Refinement

*R*[*F* ^2^ > 2σ(*F* ^2^)] = 0.027
*wR*(*F* ^2^) = 0.054
*S* = 0.85
4346 reflections
180 parameters
H atoms treated by a mixture of independent and constrained refinement
Δρ_max_ = 0.88 e Å^−3^
Δρ_min_ = −0.44 e Å^−3^
Absolute structure: Flack (1983 ▶), 1615 Friedel pairs
Flack parameter: −0.022 (7)

### 

Data collection: *CrystalClear-SM Expert* (Rigaku/MSC, 2009 ▶); cell refinement: *CrystalClear-SM Expert*; data reduction: *CrystalClear-SM Expert*; program(s) used to solve structure: *SHELXS97* (Sheldrick, 2008 ▶); program(s) used to refine structure: *SHELXL97* (Sheldrick, 2008 ▶); molecular graphics: *CrystalStructure* (Rigaku/MSC, 2009 ▶); software used to prepare material for publication: *CrystalStructure*.

## Supplementary Material

Crystal structure: contains datablocks global, I. DOI: 10.1107/S160053681002698X/pv2295sup1.cif

Structure factors: contains datablocks I. DOI: 10.1107/S160053681002698X/pv2295Isup2.hkl

Additional supplementary materials:  crystallographic information; 3D view; checkCIF report

## Figures and Tables

**Table 1:** Hydrogen-bond geometry (Å, °)

*D*—H⋯*A*	*D*—H	H⋯*A*	*D*⋯*A*	*D*—H⋯*A*
O1—H1⋯N1	0.87 (2)	1.84 (2)	2.593 (2)	143 (2)
O2—H2⋯N2^i^	0.75 (3)	2.08 (3)	2.830 (2)	173 (2)
C5—H5⋯O2^ii^	0.95	2.54	3.463 (3)	163

Symmetry codes: (i) 
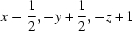
; (ii) 
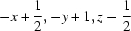
.

**Fig. 1.**
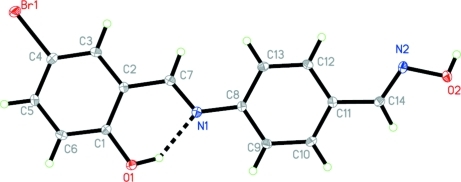
The molecule structure of the title complex with atomic numbering scheme. Displacement ellipsoids are drawn at the 30% probability level.

**Fig. 2.**
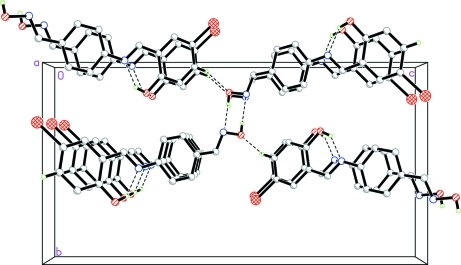
Unit cell packing of the title compound. Intramolecular and intermolecular hydrogen bonds are shown as dashed lines.

## supplementary crystallographic information

### Comment

Schiff bases have been used widely as versatile ligands in coordination chemistry (Biswas *et al.*, 2008; Dong *et al.*, 2010). Recently, the structures of a few Schiff base compounds have been reported (Dong *et al.*, 2007; Dong *et al.*, 2009). In this paper, we report the synthesis and crystal structure of the title compound, (I).

In the title compound (Fig. 1) the bond lengths and angles are in normal ranges and agree very well with the corresponding bond lengths and angles reported in similar structures (Dong *et al.*, 2007; Dong *et al.*, 2009; Zhao *et al.*, 2009). The mean planes of the two benzene rings are almost parallel to each other making a dihedral angle of 4.09 (1) ° with respect to each other. There is an intramolecular hydrogen bond, O1—H1···N1 (Tab. 1). Besides, intermolecular hydrogen bonds, O2—H2···N2 and C5—H5···O2 link molecules into infinite catenarian supramolecular shape (Tab. 1 & Fig. 2).

### Experimental

The title compound was synthesized according to methods reported earlier (Zhao *et al.*, 2009; Dong *et al.*, 2009). A solution of 1-(4-aminophenyl)-methanal (1.21 g, 10 mmol) in methanol (15 ml) was added to a mixture of hydroxylamine sulfate (1.31 g, 10 mmol) and sodium acetate (2.0 g, 25 mmol). After refluxing for 4–5 h, the reaction was completed. The solvent was evaporated under vacuo. Demineralized water (40 ml) was added, cooled to 268–265 K and filtered, resulting in 4-aminobenzaldehyde oxime as a crystalline solid (yield; 1.18 g, 86.7%; m.p. 395–397 K). To an ethalnol solution (5 ml) of 4-aminobenzaldehyde oxime (0.1362 g, 1 mmol) was added dropwise an ethanol solution (5 ml) of 5-bromo-2-hydroxybenzaldehyde (0.19995 g, 1 mmol). The mixture solution was stirred at 328–333 K for 5 h. After cooling to room temperature, the precipitate was filtered off, and washed successively three times with ethanol. The product was dried *in vacuo* and purified by recrystallization from ethanol to yield 267.5 mg (Yield, 83.8%) of solid; m.p. 490–491 K. Pale-yellow needle-like single crystals suitable for X-ray diffraction studies were obtained by slow evaporation from a solution of methanol at room temperature in about one month.

### Refinement

An absolute structure was determined by Flack (1983) method employing 1615 Friedel pairs. H atoms were treated as riding atoms with distances C—H = 0.95 Å and *U*_iso_(H) = 1.2 *U*_eq_(C). The hydroxyl H-atoms were located from a difference Fourier map and were allowed to refine freely.

### Crystal data

**Table:**

C_14_H_11_BrN_2_O_2_	*D*_x_ = 1.695 Mg m^−^^3^
*M**_r_* = 319.16	Melting point = 490–491 K
Orthorhombic, *P*2_1_2_1_2_1_	Mo *K*α radiation, λ = 0.71075 Å
Hall symbol: P 2ac 2ab	Cell parameters from 4929 reflections
*a* = 4.4279 (5) Å	θ = 1.9–32.9°
*b* = 12.1790 (16) Å	µ = 3.29 mm^−^^1^
*c* = 23.196 (2) Å	*T* = 113 K
*V* = 1250.9 (3) Å^3^	Needle, pale-yellow
*Z* = 4	0.26 × 0.24 × 0.22 mm
*F*(000) = 640	

### Data collection

**Table:**

Rigaku Saturn724 CCD diffractometer	4346 independent reflections
Radiation source: Rotating Anode	2818 reflections with *I* > 2σ(*I*)
multilayer	*R*_int_ = 0.048
Detector resolution: 14.222 pixels mm^-1^	θ_max_ = 32.9°, θ_min_ = 1.8°
ω scans	*h* = −6→6
Absorption correction: multi-scan (*CrystalClear*; Rigaku/MSC, 2009)	*k* = −18→17
*T*_min_ = 0.482, *T*_max_ = 0.532	*l* = −34→33
15336 measured reflections	

### Refinement

**Table:**

Refinement on *F*^2^	Secondary atom site location: difference Fourier map
Least-squares matrix: full	Hydrogen site location: inferred from neighbouring sites
*R*[*F*^2^ > 2σ(*F*^2^)] = 0.027	H atoms treated by a mixture of independent and constrained refinement
*wR*(*F*^2^) = 0.054	*w* = 1/[σ^2^(*F*_o_^2^) + (0.0171*P*)^2^] where *P* = (*F*_o_^2^ + 2*F*_c_^2^)/3
*S* = 0.85	(Δ/σ)_max_ = 0.001
4346 reflections	Δρ_max_ = 0.88 e Å^−^^3^
180 parameters	Δρ_min_ = −0.44 e Å^−^^3^
0 restraints	Absolute structure: Flack (1983), 1615 Friedel pairs
Primary atom site location: structure-invariant direct methods	Flack parameter: −0.022 (7)

### Special details

**Table:**

Experimental. Anal. Calc. for C_14_H_11_BrN_2_O_2_: C, 52.69; H, 3.47; N, 8.78. Found: C, 52.67; H, 3.44; N, 8.81.
Geometry. All e.s.d.'s (except the e.s.d. in the dihedral angle between two l.s. planes) are estimated using the full covariance matrix. The cell e.s.d.'s are taken into account individually in the estimation of e.s.d.'s in distances, angles and torsion angles; correlations between e.s.d.'s in cell parameters are only used when they are defined by crystal symmetry. An approximate (isotropic) treatment of cell e.s.d.'s is used for estimating e.s.d.'s involving l.s. planes.
Refinement. Refinement of *F*^2^ against ALL reflections. The weighted *R*-factor *wR* and goodness of fit *S* are based on *F*^2^, conventional *R*-factors *R* are based on *F*, with *F* set to zero for negative *F*^2^. The threshold expression of *F*^2^ > σ(*F*^2^) is used only for calculating *R*-factors(gt) *etc*. and is not relevant to the choice of reflections for refinement. *R*-factors based on *F*^2^ are statistically about twice as large as those based on *F*, and *R*- factors based on ALL data will be even larger.

### Fractional atomic coordinates and isotropic or equivalent isotropic displacement parameters (Å^2^)

**Table:**

	*x*	*y*	*z*	*U*_iso_*/*U*_eq_	
Br1	0.93894 (6)	0.310770 (17)	0.055477 (9)	0.02449 (6)	
O1	0.5216 (4)	0.65997 (11)	0.22190 (6)	0.0219 (4)	
O2	−0.9560 (4)	0.35901 (13)	0.51519 (6)	0.0228 (4)	
N1	0.1808 (4)	0.51372 (14)	0.27053 (7)	0.0158 (4)	
N2	−0.7439 (4)	0.34661 (14)	0.47039 (7)	0.0165 (4)	
C1	0.6150 (5)	0.57912 (16)	0.18617 (8)	0.0156 (5)	
C2	0.5081 (5)	0.46983 (15)	0.19213 (8)	0.0150 (5)	
C3	0.6111 (5)	0.38957 (16)	0.15310 (8)	0.0174 (5)	
H3	0.5429	0.3159	0.1565	0.021*	
C4	0.8098 (5)	0.41758 (17)	0.11007 (9)	0.0181 (5)	
C5	0.9174 (5)	0.52501 (16)	0.10466 (8)	0.0174 (4)	
H5	1.0571	0.5431	0.0751	0.021*	
C6	0.8195 (5)	0.60434 (17)	0.14244 (9)	0.0189 (5)	
H6	0.8922	0.6774	0.1387	0.023*	
C7	0.2943 (5)	0.44092 (17)	0.23670 (9)	0.0172 (5)	
H7	0.2362	0.3663	0.2411	0.021*	
C8	−0.0289 (5)	0.48808 (15)	0.31419 (8)	0.0142 (4)	
C9	−0.1308 (5)	0.57570 (17)	0.34784 (9)	0.0181 (5)	
H9	−0.0580	0.6477	0.3405	0.022*	
C10	−0.3373 (5)	0.55866 (17)	0.39193 (9)	0.0185 (5)	
H10	−0.4072	0.6195	0.4138	0.022*	
C11	−0.4435 (5)	0.45385 (15)	0.40458 (8)	0.0154 (4)	
C12	−0.3426 (5)	0.36586 (16)	0.37064 (9)	0.0175 (5)	
H12	−0.4139	0.2938	0.3783	0.021*	
C13	−0.1407 (5)	0.38292 (16)	0.32620 (8)	0.0170 (5)	
H13	−0.0766	0.3225	0.3034	0.020*	
C14	−0.6628 (5)	0.44081 (17)	0.45110 (9)	0.0171 (5)	
H14	−0.7489	0.5048	0.4678	0.021*	
H1	0.384 (5)	0.6368 (17)	0.2459 (9)	0.022 (7)*	
H2	−1.018 (7)	0.302 (2)	0.5186 (12)	0.070 (12)*	

### Atomic displacement parameters (Å^2^)

**Table:**

	*U*^11^	*U*^22^	*U*^33^	*U*^12^	*U*^13^	*U*^23^
Br1	0.03228 (13)	0.02054 (9)	0.02065 (10)	0.00167 (11)	0.00598 (11)	−0.00300 (9)
O1	0.0290 (11)	0.0162 (7)	0.0204 (8)	−0.0007 (7)	0.0061 (8)	−0.0010 (6)
O2	0.0245 (10)	0.0219 (8)	0.0220 (8)	−0.0066 (8)	0.0081 (8)	−0.0010 (6)
N1	0.0149 (10)	0.0174 (9)	0.0150 (9)	0.0026 (8)	−0.0014 (8)	0.0015 (7)
N2	0.0123 (10)	0.0234 (10)	0.0138 (8)	−0.0013 (7)	0.0002 (8)	−0.0013 (7)
C1	0.0158 (13)	0.0163 (10)	0.0146 (10)	0.0027 (9)	−0.0017 (9)	−0.0003 (8)
C2	0.0134 (14)	0.0171 (9)	0.0146 (9)	0.0017 (8)	−0.0016 (9)	0.0026 (7)
C3	0.0174 (14)	0.0151 (10)	0.0196 (11)	−0.0007 (9)	−0.0048 (10)	0.0005 (8)
C4	0.0184 (13)	0.0202 (11)	0.0157 (10)	0.0042 (9)	−0.0025 (10)	−0.0015 (9)
C5	0.0149 (12)	0.0215 (10)	0.0157 (10)	0.0002 (10)	0.0016 (11)	0.0023 (8)
C6	0.0217 (13)	0.0148 (10)	0.0201 (11)	−0.0032 (9)	−0.0022 (10)	0.0014 (8)
C7	0.0159 (12)	0.0164 (10)	0.0194 (11)	−0.0005 (9)	−0.0019 (10)	0.0015 (9)
C8	0.0112 (12)	0.0177 (9)	0.0136 (9)	−0.0004 (9)	−0.0016 (9)	0.0009 (7)
C9	0.0186 (14)	0.0149 (10)	0.0209 (11)	0.0001 (9)	0.0010 (10)	0.0005 (8)
C10	0.0210 (13)	0.0168 (10)	0.0178 (11)	0.0031 (9)	−0.0005 (10)	−0.0037 (8)
C11	0.0129 (11)	0.0199 (10)	0.0134 (9)	−0.0015 (10)	−0.0030 (10)	−0.0001 (7)
C12	0.0195 (13)	0.0140 (10)	0.0190 (10)	0.0003 (9)	−0.0009 (10)	0.0027 (8)
C13	0.0189 (14)	0.0151 (10)	0.0170 (10)	0.0028 (9)	−0.0033 (9)	−0.0012 (8)
C14	0.0161 (11)	0.0184 (10)	0.0169 (11)	0.0001 (8)	−0.0019 (10)	−0.0024 (9)

### Geometric parameters (Å, °)

**Table:**

Br1—C4	1.903 (2)	C5—H5	0.9500
O1—C1	1.352 (2)	C6—H6	0.9500
O1—H1	0.87 (2)	C7—H7	0.9500
O2—N2	1.409 (2)	C8—C9	1.397 (3)
O2—H2	0.75 (3)	C8—C13	1.401 (3)
N1—C7	1.286 (2)	C9—C10	1.387 (3)
N1—C8	1.409 (3)	C9—H9	0.9500
N2—C14	1.283 (2)	C10—C11	1.392 (3)
C1—C6	1.394 (3)	C10—H10	0.9500
C1—C2	1.419 (3)	C11—C12	1.403 (3)
C2—C3	1.408 (3)	C11—C14	1.460 (3)
C2—C7	1.445 (3)	C12—C13	1.380 (3)
C3—C4	1.374 (3)	C12—H12	0.9500
C3—H3	0.9500	C13—H13	0.9500
C4—C5	1.398 (3)	C14—H14	0.9500
C5—C6	1.375 (3)		
			
C1—O1—H1	111.7 (14)	N1—C7—H7	119.2
N2—O2—H2	103 (2)	C2—C7—H7	119.2
C7—N1—C8	122.91 (18)	C9—C8—C13	118.23 (19)
C14—N2—O2	110.38 (17)	C9—C8—N1	116.44 (18)
O1—C1—C6	118.97 (18)	C13—C8—N1	125.33 (18)
O1—C1—C2	121.43 (19)	C10—C9—C8	120.70 (19)
C6—C1—C2	119.60 (18)	C10—C9—H9	119.6
C3—C2—C1	118.72 (19)	C8—C9—H9	119.6
C3—C2—C7	120.18 (18)	C9—C10—C11	121.0 (2)
C1—C2—C7	121.09 (18)	C9—C10—H10	119.5
C4—C3—C2	120.14 (19)	C11—C10—H10	119.5
C4—C3—H3	119.9	C10—C11—C12	118.3 (2)
C2—C3—H3	119.9	C10—C11—C14	118.71 (18)
C3—C4—C5	121.05 (19)	C12—C11—C14	122.92 (18)
C3—C4—Br1	120.41 (16)	C13—C12—C11	120.70 (19)
C5—C4—Br1	118.53 (16)	C13—C12—H12	119.7
C6—C5—C4	119.5 (2)	C11—C12—H12	119.7
C6—C5—H5	120.2	C12—C13—C8	120.99 (19)
C4—C5—H5	120.2	C12—C13—H13	119.5
C5—C6—C1	120.93 (19)	C8—C13—H13	119.5
C5—C6—H6	119.5	N2—C14—C11	122.78 (18)
C1—C6—H6	119.5	N2—C14—H14	118.6
N1—C7—C2	121.63 (19)	C11—C14—H14	118.6
			
O1—C1—C2—C3	179.22 (19)	C7—N1—C8—C9	179.5 (2)
C6—C1—C2—C3	−0.5 (3)	C7—N1—C8—C13	−0.4 (3)
O1—C1—C2—C7	0.1 (3)	C13—C8—C9—C10	0.0 (3)
C6—C1—C2—C7	−179.5 (2)	N1—C8—C9—C10	−179.95 (19)
C1—C2—C3—C4	−0.3 (3)	C8—C9—C10—C11	1.3 (3)
C7—C2—C3—C4	178.75 (19)	C9—C10—C11—C12	−1.6 (3)
C2—C3—C4—C5	1.0 (3)	C9—C10—C11—C14	−179.44 (19)
C2—C3—C4—Br1	−177.75 (16)	C10—C11—C12—C13	0.6 (3)
C3—C4—C5—C6	−1.0 (3)	C14—C11—C12—C13	178.34 (19)
Br1—C4—C5—C6	177.87 (17)	C11—C12—C13—C8	0.7 (3)
C4—C5—C6—C1	0.1 (3)	C9—C8—C13—C12	−1.0 (3)
O1—C1—C6—C5	−179.1 (2)	N1—C8—C13—C12	178.95 (19)
C2—C1—C6—C5	0.5 (3)	O2—N2—C14—C11	179.74 (18)
C8—N1—C7—C2	179.75 (19)	C10—C11—C14—N2	−171.1 (2)
C3—C2—C7—N1	−175.3 (2)	C12—C11—C14—N2	11.2 (3)
C1—C2—C7—N1	3.8 (3)		

### Hydrogen-bond geometry (Å, °)

**Table:**

*D*—H···*A*	*D*—H	H···*A*	*D*···*A*	*D*—H···*A*
O1—H1···N1	0.87 (2)	1.84 (2)	2.593 (2)	143 (2)
O2—H2···N2^i^	0.75 (3)	2.08 (3)	2.830 (2)	173 (2)
C5—H5···O2^ii^	0.95	2.54	3.463 (3)	163

Symmetry codes: (i) *x*−1/2, −*y*+1/2, −*z*+1; (ii) −*x*+1/2, −*y*+1, *z*−1/2.

## References

[bb1] Biswas, C., Drew, M. G. B. & Ghosh, A. (2008). *Inorg. Chem.* **47**, 4513–4519. 10.1021/ic8002542 18459727

[bb2] Dong, W.-K., Duan, J.-G., Dong, C.-M., Ren, Z.-L. & Shi, J.-Y. (2007). *Z. Kristallogr. New Cryst. Struct.* **222**, 327–328.

[bb3] Dong, W.-K., He, X.-N., Yan, H.-B., Lv, Z.-W., Chen, X., Zhao, C.-Y. & Tang, X.-L. (2009). *Polyhedron*, **28**, 1419–1428.

[bb4] Dong, W.-K., Sun, Y.-X., Zhao, C.-Y., Dong, X.-Y. & Xu, L. (2010). *Polyhedron*, **29**, 2087–2097.

[bb5] Flack, H. D. (1983). *Acta Cryst.* A**39**, 876–881.

[bb6] Rigaku/MSC (2009). *CrystalClear*, *CrystalClear-SM Expert* and *CrystalStructure* Rigaku/MSC, The Woodlands, Texas, USA.

[bb7] Sheldrick, G. M. (2008). *Acta Cryst.* A**64**, 112–122. 10.1107/S0108767307043930 18156677

[bb8] Zhao, L., Dong, W.-K., Wu, J.-C., Sun, Y.-X. & Xu, L. (2009). *Acta Cryst.* E**65**, o2462. 10.1107/S1600536809033753 PMC2970305 21577917

